# Use of Calcium Amino Acid Chelate in the Production of Acid-Curd Goat Cheese

**DOI:** 10.3390/foods9080994

**Published:** 2020-07-24

**Authors:** Małgorzata Pawlos, Agata Znamirowska, Grzegorz Zaguła, Magdalena Buniowska

**Affiliations:** 1 Department of Dairy Technology, Institute of Food Technology and Nutrition, University of Rzeszow, Ćwiklińskiej 2D, 35601 Rzeszów, Poland; aznam@univ.rzeszow.pl; 2 Department of Bioenergetics, Food Analysis and Microbiology, Institute of Food Technology and Nutrition, University of Rzeszow, Ćwiklińskiej 2D, 35601 Rzeszów, Poland; g_zagula@univ.rzeszow.pl

**Keywords:** acid-curd cheese, goat’s milk, whey, calcium amino acid chelate, mineral composition, organoleptic properties, texture

## Abstract

Amino acid chelates are a new group of compounds approved for food enrichment, however there is no previous research using calcium amino acid chelate to enrich goat’s milk products. The purpose of this research was to evaluate the possibility of using calcium amino acid chelate to produce goat’s acid-curd cheese. In this study, four types of acid-curd cheeses from goat’s milk subjected to 85 °C/5 min treatment were produced: control cheeses—made from milk without calcium addition and cheeses from milk enriched with 30, 35 and 40 mg of Ca (in 100 g of milk) in the form of calcium amino acid chelate. Goat cheese with calcium amino acid chelate had a higher moisture content, and a lower fat content. More fat was separated with the whey. In cheeses made from the milk with calcium amino acid chelate there was no goaty taste. Enrichment with 35 mg of Ca in 100 g of goat milk increased the calcium content in cheese by 60.5% in comparison to the control sample. However, the enrichment of goat milk with 40 mg Ca (in 100 g of processed milk) increased the calcium content in cheese by only 63.29%.

## 1. Introduction

According to Clark and Mora Garcia [1] and Park et al. [2] caprine milk is characterized by enhanced digestibility, better alkalinity, buffering capacity and is beneficial to human health in comparison with cow milk. Goat’s milk is often suggested as a substitute of cow’s milk for allergic patients, because of a lower quantity of α-caseins, but the results about its cross-reactivity are still controversial [3]. According to Monaci et al. [4] milk of all ruminant species contains homologous proteins, which share the same properties and contribute to the cross-reactivity phenomenon in allergic individuals. Higher levels of calcium, potassium, iron, copper and manganese are found in goat’s milk than in cow’s milk and the content of these elements is higher compared to cow’s milk [5]. However, the chemical composition of milk including the macro- and microelements content is not constant and depends on a variety of environmental, genetics and physiological factors [5,6].

Since ancient times goat milk has been used for manufacture of different types of cheeses worldwide [7]. Acid-curd cheeses are a characteristic dairy products in the countries of Central and Eastern Europe. In East-Central Europe Tvarog is widely available and a well-known and valued group of traditional fresh acid-curd cheeses. Acid curd cheese from whole milk in the daily diet is a valuable source of high biological value protein, easily digestible fat, vitamins and minerals [8]. In the production of acid-curd cheese, two methods of coagulation of milk proteins are used: acid or acid-rennet. According to Danków and Pikul [6], Bonfim da Silva and Pereira da Costa [9] and Domagała [10] the acid curd from goat milk has a very delicate structure, less firmness and viscosity compared to cow’s milk curd. Additionally, it is easy to brittle, which makes it difficult to process. Miocinovic et al. [11] study showed that the differences in the rheological properties of acid gels obtained from cow and goat milk are a consequence of milk’s different composition and properties. The lower texture and viscosity properties of fermented goat’s milk products are the result of their fragile microstructure, less resistant and sensitive to fast deformation and to the less compact, soft and weak acid gels [10,12]. According to Delgado et al. [12] and Park [13] these properties of goat’s milk are related with the lower mean diameter, degree of hydration, mineralization and αs_1_-casein content in milk and with the smallest diameter of non-protein nitrogen in goat milk in relation to cow milk.

An essential role of calcium ions during the aggregation phase of coagulation process is the interconnection of caseins with the help of calcium bridges. On heat treatment of milk, a portion of the calcium ions is transformed into insoluble calcium phosphate, which is not involved in coagulation processes. Calcium chloride is often added to milk before pasteurization process to restore gel-formation capability [14,15].

According to Szajnar et al. [16] milk and dairy products are the most important sources of calcium intake in a human diet. Dietary calcium absorption from food reaches from 10% to 40% and nearly 32% of Ca is absorbed from milk and dairy products [16,17,18]. The high bioavailability of calcium from dairy products is due to both the ratio of calcium to phosphorus and the presence of numerous components that increase absorption (vitamin D, phosphopeptides released during casein hydrolysis, l-lysine, l-arginine, lactose) [19,20]. The amount of protein consumed affects the body’s calcium balance. The adverse effect of protein on calcium utilization occurs when the ratio of consumed calcium to protein is below 20. For acid curd cheeses, the calcium to protein ratio is between 7 and 12 [21]. Acid-curd cheese is characterized by a lower calcium content in relation to phosphorus, which also contributes to lowering of calcium absorption. This unfavorable calcium to phosphorus ratio is a consequence of acid-curd cheese production technology [20]. Acidification process results in the dissociation of colloidal calcium phosphate which is the main reason for lower calcium content in cheeses coagulated by acidification [22]. During separation of the whey from the cheese curd, up to 80% of the amount of calcium from the milk being processed is lost [20]. A different method that allows to increase the protein utilization in this type of cheese is the calcium-heat-acid coagulation method. It is performed by enriching milk with calcium ions, high pasteurization and coagulation of proteins by the acid method [23,24]. According to Szpendowski et al. [24] during milk heating, the complexes are formed between β-lactoglobulin and α-lactalbumin and casein via disulfide, hydrogen and ionic bonds, in which amorphous calcium phosphate is involved. Whey proteins form stable complexes, mainly with α_s1_-casein, β-casein and κ-casein fractions. Acid curd cheese made from milk enriched with calcium ions and exposed to high pasteurization has a higher nutritional value of protein in comparison with cheese produced without calcium addition. These studies were performed using cow’s milk, which has a higher heat stability than goat milk [25]. Whey proteins have a higher nutritional value than caseins and the higher nutritional value of cheese produced by calcium-heat-acid coagulation method is partly caused by the denatured whey proteins becoming an integrated part of the cheese [26].

Losses of the ionic calcium during milk pasteurization may be reduces by addition of calcium compounds (lactate, gluconate, chloride) to the milk before pasteurization. Such compounds will acidify the milk therefore increase the risk of protein coagulation during pasteurization [27]. Addition of calcium amino acid chelate will, however, make the milk alkaline. This amino acid chelate consists of glycine and calcium. The ionic ratio of calcium to glycine is 1:2 [28]. Amino acid chelates are a new group of compounds approved for food fortification, however there is no previous research using calcium amino acid chelate to enrich goat’s milk and goat’s milk products. A priority in the enrichment of dairy products is the appropriate selection of the compounds that are carriers of minerals. Moreover, safety and availability for the human body are the features that should be characterized by chemical substances used as carriers of minerals [29]. According to Commission Regulation (EC) No. 953/2009 of 13 October 2009 [30] and Commission Regulation (EC) No. 1170/2009 of 30 November 2009 [31] calcium amino acid chelate is commonly used in the manufacture of food supplements and substances that may be added for specific nutritional purposes.

The aim of this research was to evaluate the possibility of using calcium amino acid chelate to produce fresh acid-curd cheese from goat’s milk and determine the migration of mineral compounds from milk to cheese and whey. Moreover, the effect of amino acid chelates on the physical, chemical and organoleptic characteristics of these cheeses and physicochemical properties of whey was evaluated.

## 2. Materials and Methods

### 2.1. Raw Goat’s Milk Analysis

Raw caprine milk for the production of fresh acid-curd cheese was collected in July directly from an organic farm „Zuza” in Zabratówka village (Podkarpacie region, Poland), from different coloured goats of mixed breeds. The physicochemical and microbiological analysis of the chilled (4 °C) morning milk samples was performed before cheese manufacture. The total bacterial count (TBC) and somatic cell count (SCC) were determined in BactoCount IBC M/SCC semi-automatic counter (Bentley Instruments Inc., Chaska, Minnesota, USA). The chemical composition (protein, fat, lactose, total solids) and freezing point were determined in milk and milk products composition analyzer Bentley B-150 (Bentley Instruments Inc., Chaska, Minnesota, USA) which is based on molecular-bond energy absorptions in the mid-infrared spectrum. The determination of casein was performed by the formol method according to Scott et al. [32], titratable acidity—according to Calamari et al. [33] and density of goat milk was performed in temperature 20 °C according to Raţu et al. [34]. pH value was determined with a digital pH meter Toledo FiveEasy TM (Mettler Toledo, Greifensee, Switzerland).

### 2.2. Cheese Manufacture

After filtration to remove dirt and foreign particles the goat milk was divided into four batches: (a) CM—control milk without calcium addition, (b) M30—with 30 mg of Ca in 100 g of milk, (c) M35—with 35 mg of Ca in 100 g of milk, (d) M40—with 40 mg of Ca in 100 g of milk and thoroughly mixed. Calcium was added as calcium amino acid chelate Albion^®^ with 26% of Ca concentration (Olimp Laboratories, Dębica, Poland). Each cheese was produced from 5000 g of milk. Control milk and milk enriched with calcium amino acid chelate were pasteurized (85 °C, 5 min). After the heat treatment milk was cooled to 28 °C and each batch of milk was inoculated with 0.20% (w/w) starter culture of mesophilic lactic fermentation bacteria G500—*Lactococcus lactis* subsp. *lactis, Lactococcus lactis* subsp. *cremoris, Lactococcus lactis* subsp. *lactis* biovar *diacetylactis* (CSK Food Enrichment, Wageningen, The Netherlands), 15 min prior to addition of 0.02% (v/w) of Beaugel 5 rennet (Coquard, Villefranche sur Saône, France). The rennet strength was 1/3000 with 150 mg of active chymosin per liter. Then the milk was gently mixed and incubated at 28 °C, until the pH reached 4.6 (±0.50) value (approx. 14–16 h). The obtained curds were cut into a cube (15 × 15 × 15 mm), heated in water bath in temperature 35 °C for 1.5 h. The temperature was increased to 40 °C for 0.5 h. Next, the whey was separated with a cheesecloth and collected in a coded glass beakers (CW—control from CM milk, W30—whey from M30, W35—whey from M35, W40—whey from M40) and curds were transferred to molds and pressed (10 N kg^−1^, 0.5 h) in a press with pneumatic cylinders (Pneumatig, Gdynia, Poland). Acid-curd cheese was cooled to 6 °C and stored in individual plastic containers at the same temperature for 24 h, after which analysis was carried out. Cheese was coded: CC—control cheese from CM milk, C30—cheese from M30 milk, C35—cheese from M35 milk, C40—cheese from M40 milk.

### 2.3. Physical and Chemical Parameters of Cheese

All physicochemical analyses were run on ground cheese samples to achieve uniformity. The determination of pH was carried out by a digital pH meter Toledo FiveEasy TM (Metler Toledo, Greifensee, Switzerland). Titratable acidity was determined in °SH [34]. The fat content was measured according to the Gerber method [35]. The moisture content was calculated using moisture analyzer MA 50.R (Radwag, Radom, Poland), according to Kowalska et al. [36] method. The content of protein in the milk and whey was used to calculate the degree of retention of protein in the cheese, according to Siemianowski et al. [37].

### 2.4. Acid-Curd Cheese Texture Analysis

Texture profile analysis (TPA) was performed with Brookfield CT3 texture analyzer (Brookfield AMETEK, Middleboro, Massachusetts, USA), controlled by a PC computer, according to Pawlos et al. [38]. With modifications. Cheese samples were cut into a cube (20 × 20 × 20 mm) and stored at a temperature 10 °C ± 1 °C before testing. Penetration testing was carried out using a plastic plunger type TA3/100 (25.4 mm diameter) to the depth of 15 mm at a rate of 5 mm s^−1^ and press force 1.00 N. The hold time between the first and second compression was 1 s. TPA was used to measure hardness (N), adhesiveness (mJ), cohesiveness and springiness (mm).

### 2.5. Organoleptic Evaluation of Acid-Curd Cheese

Organoleptic evaluation of cheese was carried out according to the procedure of International Standards ISO4121:2003 [39]. Twenty panelists were selected to evaluate appearance, consistency, taste, odour and overall acceptability on a hedonic scale from 5 to 1 (5—extremely liked, 4—moderately liked, 3—neither liked nor disliked, 2—moderately disliked, 1—extremely disliked).

### 2.6. Whey Analysis

The determination of pH was carried out by a digital pH meter Toledo FiveEasy TM (Mettler Toledo, Greifensee, Switzerland). Titratable acidity (°SH) was determined according to Calamari et al. [33]. The chemical composition of whey (protein, fat, lactose) was determined in Bentley B-150 analyzer (Bentley Instruments Inc., Chaska, Minnesota, USA).

### 2.7. Mineral Composition of Milk, Cheese and Whey

The concentration of macro- and microelements (Ca, K, Mg, P, Mn, Se, Cd and Pb) in milk, cheese and whey was determined by ICP-OES (inductively coupled plasma—optical emission spectrometry) method with a spectrophotometer Thermo iCAP Dual 6500 (Thermo Fisher Scientific Inc., USA) according to Znamirowska et al. [40] with modifications (2.50 g of milk, 1.00 g of whey and 0.50 g of cheese were weighed). The ICP-OES instrument was calibrated with standards (Merck, Darmstadt, Germany) including concentrations of 10,000 ppm for macroelements and 1000 ppm for microelements. The Certified Reference Material (CRM) was used for validation of analytical methods and the recovery obtained for Ca was 98.00%, for K—99.00%, for Mg—101.00%, for P—98.00%, for Mn—102.00%, for Se—102.00%, for Cd—102.00% and for Pb—97.00%.

### 2.8. Statistical Analysis

Statistical analysis was performed according to Pawlos et al. [38]. From the obtained results the mean, standard deviation and simple correlation coefficient *r* were worked out statistically in the software Statistica 13.1 (StatSoft, Tulsa, Oklahoma, USA). A one-way analysis of ANOVA variance was performed and significance of differences between the averages (*p* ≤ 0.05) was estimated with Tukey’s test. The experiment was repeated three times on different occasions. Four samples were tested for each cheese and whey variant and it was repeated for three cheese making trials.

## 3. Results

### 3.1. Quality of Raw Goat’s Milk

The composition and physicochemical characteristic of raw goat’s milk is presented in Table 1. The protein, fat and total solids values were higher than those reported by Barłowska et al. [5], Wolanciuk et al. [41] and Znamirowska et al. [40] for White improved goat breed milk and milk from unidentified goats. The gross chemical composition was, however, in accordance with that reported by Guo et al. [42] for commingled goat milk. The titratable acidity and pH of goat milk was in accordance with results obtained by Domagała and Wszołek [43], Park et al. [2], Strzałkowska et al. [44].

The microbiological and hygienic quality of raw milk were evaluated by TBC and SCC [45,46]. The SCC criteria for goat’s milk were not defined in European Commision Regulation No. 1662/2006 amending Regulation (EC) No. 853/2004 of the European Parliament and of the Council [47] in the same way as SCC for cow’s milk [45,48,49].Chen et al. [50] divided goat milk into three groups depending on SCC level: <500,000 (low), 500,000 to 1,000,000 (medium), and 1,000,000 to 1,500,000 (high) cells/mL. According to the European Commision Regulation No 1662/2006 [47] in raw goat milk intended for the production of thermized milk or other milk products, the presence of up to 1.5 million of microorganisms in 1 mL is allowed and for products from raw milk the TBC may not exceed 500 000 cfu/mL. According to Park and Haenlein et al. [51] goat milk is produced mainly by apocrine secretion, which results in a large number of cytoplasmic particles (similar in size to somatic cells) and epithelial cells in. Bagnicka et al. [52] reported that about 33% of total SCC in healthy goats’ mammary gland is due to cytoplasmic particles along with fat. Cytoplasmic particles and epithelial cells are, however, not fully differentiated while using an automatic cell counter instead of staining methods [53]. Also for this reason, SCC could be overstated in our research. TBC above 500,000 cfu mL^−1^ negatively change the quality of milk, when values below 500,000 cfu mL^−1^ are considered as an indicator of acceptable quality of milk [54,55]. In this study, TBC was below 500,000 cfu mL^−1^ and SCC was lower than 1 million mL^−1^, which indicates acceptable microbiological and cytological quality of the milk.

The milk was characterized by a high content of K, P, Ca and Mg (Table 2). Similar content of macroelements was found by Bergillos-Meca et al. [56] for goat milk. The study of Park et al. [2] showed a lower content of Ca, P, K and Mn and a higher Mg and Se content in goat milk compared to our result. Pastuszka et al. [57] determined 189.6 mg 100 g^−1^ of P, 12.64 mg 100 g^−1^ of Mg and 8.4 µg 100 g^−1^ of Mn in goat’s milk. In our study no presence of Cd and Pb was detected in the tested milk.

### 3.2. Mineral Composition of Acid-Curd Cheese

The mineral composition of the cheeses is shown in Table 3. Enrichment of milk with calcium amino acid chelate resulted in a significant increase in calcium content in cheese (*r* = 0.9825, *p* < 0.05). An enrichment of 100 g of goat milk with a 30 mg of Ca resulted in a higher calcium content by 44.54 mg 100 g^−1^ in C30 cheese in compared to the control cheese. Increasing the calcium dose to 35 mg in 100 g of goat milk increased the amount of Ca in C35 cheese by 95.2 mg 100 g^−1^. Therefore, the enrichment of goat milk with 40 mg of Ca increased the Ca content in cheese by only 99.58 mg compared to CC cheese. The goat milk used in this study had a high K content, reflected in a high K content in the cheese. According to Raynal-Ljutovac et al. [58] concentrations of K and Mg in cheese, which are basically soluble, decrease as dry matter increases during pressing or ageing process. Control cheese showed a higher Ca content by approx.16 mg 100 g^−1^ than the goat cheese in study by Raynal-Ljutovac et al. [58] and 29 mg 100 g^−1^ higher than values published by Baran et al. [59]. Barłowska et al. [5] and Pastuszka et al. [57] confirms that goat’s milk and its products are a valuable source of K, Ca, Cu and Mn. The enrichment of milk with Ca did not significantly affect the content of Mg, P, Mn and Se in the cheeses. In the research of Baran et al. [59] a lower Mg content was found in acidic goat cheeses (11.94 mg 100 g^−1^), while higher content was observed in goat acidic-rennet cheeses (19.29 mg 100 g^−1^) compared to our cheeses. In all goat cheeses the selenium content was 1 µg 100 g^−1^. According to Allen and Miller [60], most of selenium remains in the unbound ionic form in goat milk and does not bind to proteins at normal pH of milk. However, when the pH value decreases below 6.0, a part of the selenium may be bound by the casein fraction. Our studies do not confirm this observation. Selenium is absorbed by the plants from the soil and then converted into organic forms of this element, which are incorporated into proteins [61]. According to Pizzoferrato [62] this element is concentrated in cheese by the drying (ripening) effect. As in milk, Cd and Pb were not found in cheeses either.

### 3.3. Physicochemical Properties and Texture of Cheese

Table 4 presents the acidity, chemical composition and selected texture parameters of fresh goat cheeses as a function of on the quantity of calcium added to the processed milk. Enrichment of goat’s milk with calcium amino acid chelate causes alkalization of milk before fermentation [16]. This phenomenon was also observed in the present study, because in cheese C30, C35 and C40 a significantly higher pH value and lower titratable acidity were determined in comparison with CC cheese. These relationships were confirmed by significant correlation coefficients. Janštová et al. [63] showed that the pH value in fresh goat cheeses was in the range from 4.75 to 5.12. Cankurt [64] determined the pH in the range from 4.81 to 4.94 in white cheese on the first day of storage. However, in the studies of Asteri et al. [65] the pH of fresh goat cheeses on the first day of storage was 4.65.

Considering the content of water, fat and protein retention in fresh goat cheese, depending on the amount of calcium added to milk, a higher protein retention and water content in C30, C35, C40 cheese were found compared to CC cheese. The increase of protein content in cheeses made from milk enriched with calcium amino acid chelate could be the result of the interaction between casein and whey proteins during milk high-pasteurization thermal process. A significantly higher moisture content and lower fat content was found in cheeses with calcium amino acid chelate. Control cheese had the lowest water content, which resulted in the highest hardness, springiness and cohesion (Table 4). Moreover, the increase of moisture content and decrease of hardness of acid-curd cheese with calcium addition could affect the cheese yield.

Enrichment of milk with calcium amino acid chelate significantly reduced cheese hardness with exception of cheese from C30. With increasing calcium dose, the hardness decreased, which is connected with increased amount of moisture in the cheese (*r* = −0.8285, *p* < 0.05). A stronger correlation was calculated between the calcium dose and cheese springiness (*r* = −0.9441, *p* < 0.05). According to Walia et al. [66] the adhesiveness definition is “the force needed to displace the material that adheres to the mouth during eating”. In this study, it was shown that cheese made from calcium enriched milk had higher adhesiveness than control cheese. A significant correlation coefficient was also calculated (*r* = −0.9395, *p* < 0.05), which confirms the effect of enrichment with calcium on cheese adhesiveness.

### 3.4. Organoleptic Evaluation of Acid-Curd Cheese

An organoleptic evaluation of cheese was carried out and the results are presented in Table 5. CC cheese was characterized by an even white colour, milky and slightly acidic taste, goaty taste, fermentation odour with perceptible diacetyl odour and a homogeneous consistency. The control cheese was the most acceptable and the panelists gave it the highest scores. During the appearance evaluation, the colour of the acid-curd cheeses was also taken into consideration. Cheeses produced from milk enriched with calcium amino acid chelate were characterized by a darker colour resulting from the gray colour of the Albion additive. Cheeses with calcium amino acid chelate were less acidic and had a low perceptible fermentation odour, which could be related to higher pH value and lower titratable acidity (Table 4). In cheeses made from the milk enriched with calcium amino acid chelate no goaty taste were observed, which was the case in the control cheeses. Znamirowska et al. [67] study showed that the addition of mineral compounds reduced the intensity of goat and sour taste in goat milk yoghurts during 21 days of cold storage. Cheeses with calcium amino acid chelate had a softer and more smeary texture than the control cheese. Softness of the cheese is also confirmed by the results of instrumental texture measurements, as the hardness in cheeses with calcium amino acid chelate was significantly lower than in control cheese. In cheeses with calcium amino acid chelate, a higher water content and relatively less fat was found, and these parameters shape the consistency of the cheeses. The compounds responsible for goaty flavour have been attributed to the presence of 4-methyloctanoic, 4-ethyloctanoic, hexanoic, octanoic, nonanoic and decanoic acids [68,69]. Previous research indicates, however, that there are more than 80 compounds which determine the fresh goat cheese flavour [70]. These include neutral basic compounds, like aldehydes, ketones, heat-induced sugar products, lactones, and animal/fecal compounds together with various fatty acids. Moreover, the “goaty” taste is also related to the presence of short-chain fatty acids (caproic, caprylic, and capric acids), which have higher concentrations than in the cow’s milk [2,71].

### 3.5. Physicochemical Properties of Whey

Whey presents 80–90% of the volume of milk for cheese making and is the major by-product. According to Moreno-Indias et al. [72] and Yang et al. [73] whey from acid cheese production contains about 4.5% (*w*/*v*) lactose, 0.8% (*w*/*v*) protein, 1% (*w*/*v*) salts, and 0.1 to 0.8% (*w*/*v*) lactic acid. The physicochemical properties of goat acid whey are presented in Table 6. The lowest pH value was determined in the control whey (CW), while in the whey W30. W35 and W40 the pH was significantly higher. However, the inverse relationship was observed for titratable acidity. The acid whey obtained in this research was characterized by a low content of protein and with increasing calcium enrichment a low concentration of protein was observed (*r* = −0.9173, *p* < 0.05). Some of the whey proteins have been attached to casein and remained in the cheese. Enrichment with calcium amino acid chelate increased the fat content in the whey and reduced the concentration of fat in the cheese. The fat content in the whey varied from 0.25 g 100 g^−1^ (CW) to 0.60 g 100 g^−1^ (W40) which are in accordance with Popović-Vranješ et al. [74]. Considering the lactose content in acid whey, it should be stated that adding of calcium amino acid chelate had a beneficial effect on lactose fermentation level in whey W40. This probably indicates that calcium amino acid chelate stimulated the growth of mesophilic bacteria that used lactose for fermentation.

### 3.6. Mineral Composition of Whey

Table 7 presents the mineral composition of acid whey obtained during the cheese production from goat milk enriched with calcium amino acid chelate. The calcium concentration in whey increased with the increasing dose of calcium amino acid chelate (*r* = 0.8939, *p* < 0.05). It shows that only part of the calcium was bound to the micelles and remained in the cheese. The concentration of K and Mg were higher than those obtained by Baran et al. [59], who determined in goat acid whey 162.65 mg 100 g^−1^ of K and 17.29 mg 100 g^−1^ of Mg. According to Raynal-Ljutovac et al. [58] during the production of cheese Rocamadour, Ste Maure and Babia Laciana the strong and early decrease in pH appearing throughout coagulation make Ca, P and Zn (mainly bound to caseins) soluble and is lost in the whey during the draining process. In our studies, goat whey contained small amounts of P and Mn, while Se was determined only in whey W35 and W40 enriched with calcium amino acid chelate.

## 4. Conclusions

Calcium amino acid chelate may be used to enrich goat’s milk to produce fresh acid-curd cheese. The addition of calcium amino acid chelate does not cause acidification of the milk but a slight alkalization, which increases the thermal stability of goat milk proteins. Fresh acid-curd goat cheese with calcium amino acid chelate had a higher protein retention, a higher moisture content and lower fat content. More fat was separated with the whey when calcium amino acid chelate was added to the milk. Enrichment with 30 mg of Ca in 100 g of goat milk increased the calcium content in cheese by 28% in comparison to the control sample. Increasing the calcium dose to 35 mg (in 100 g of processed milk) increased the calcium concentration in cheese by 60.5%. However, the enrichment of goat milk with 40 mg Ca (in 100 g of processed milk) increased the calcium content in cheese by only 63.29%. Enrichment with calcium amino acid chelate did not significantly influenced the content of K, Mg, P and Mn in cheeses and whey. In cheeses made from the milk with calcium amino acid chelate there was no goaty taste, which was perceptible in the control cheese. The cheeses with calcium addition also had a softer and more smeary consistency. Reducing the intensity of goaty and sour taste by the application of calcium amino acid chelate is very important information for goat’s milk processing industry.

## Figures and Tables

**Table 1:** Composition and physicochemical characteristic of raw goat’s milk.

Properties	Mean ± SD ^1^	Min ^2^ − Max ^3^
TBC ^4^, cfu mL^−1^	269,800 ± 17,310	249,600 − 291,120
SCC ^5^, in 1 mL	910,000 ± 32,840	870,310 − 943,940
Density, g mL^−1^	1.030 ± 0.01	1.029 − 1.031
Freezing point, °C	−0.574 ± 0.05	−0.634 − 0.524
pH	6.62 ± 0.03	6.59 − 6.68
Titratable acidity, °SH	6.70 ± 0.05	6.63 − 6.76
Protein, g 100 g^−1^	4.02 ± 0.04	3.98 − 4.08
Casein, g 100 g^−1^	3.29 ± 0.02	3.25 − 3.31
Fat, g 100 g^−1^	4.03 ± 0.07	3.95 − 4.12
Lactose, g 100 g^−1^	4.49 ± 0.09	4.39 − 4.59
Total solids, g 100 g^−1^	12.98 ± 0.01	12.95 − 12.99

^1^ SD—standard deviation. ^2^ Min—minimum value. ^3^ Max—maximum value. ^4^ TBC—total bacterial count. ^5^ SCC—somatic cell count. *n* = 12.

**Table 2:** Mineral composition of raw goat’s milk.

Mineral Compound	Mean ± SD ^1^	Min ^2^ − Max ^3^
Ca, mg 100 g^−1^	183.57 ± 0.72	180.90 − 184.52
K, mg 100 g^−1^	220.68 ± 1.07	219.01 − 222.00
Mg, mg 100 g^−1^	14.61 ± 0.04	14.55 − 14.65
P, mg 100 g^−1^	134.71 ± 3.91	130.80 − 139.54
Mn, µg 100 g^−1^	6.00 ± 1.00	5.00 − 7.00
Se, µg 100 g^−1^	2.00 ± 1.50	0.49 − 3.50
Cd, mg 100 g^−1^	ND ^4^	-
Pb, mg 100 g^−1^	ND ^4^	-

^1^ SD—standard deviation. ^2^ Min—minimum value. ^3^ Max—maximum value. ^4^ ND—not detected. *n* = 12.

**Table 3:** Mineral composition of acid-curd cheese.

Mineral Compound	Cheese Type				*r*
	CC	C30	C35	C40	
Ca, mg 100 g^−1^	157.34 ^a^ ± 1.17	201.88 ^b^ ± 1.65	252.54 ^c^ ± 5.06	256.58 ^c^ ± 0.32	0.9825 *
K, mg 100 g^−1^	174.42 ^a^ ± 1.57	175.94 ^a^ ± 1.22	175.83 ^a^ ± 0.67	175.57 ^a^ ± 2.37	0.0710
Mg, mg 100 g^−1^	19.70 ^a^ ± 0.84	19.89 ^a^ ± 0.74	20.50 ^a^ ± 0.75	20.83 ^a^ ± 0.88	0.5843
P, mg 100 g^−1^	161.43 ^a^ ± 2.62	168.57 ^a^ ± 3.17	167.98 ^a^ ± 1.25	164.46 ^a^ ± 2.56	0.5894
Mn, µg 100 g^−1^	6.00 ^a^ ± 2.00	6.00 ^a^ ± 2.00	6.00 ^a^ ± 3.00	6.00 ^a^ ± 3.00	−0.0399
Se, µg 100 g^−1^	1.00 ^a^ ± 1.00	1.00 ^a^ ± 1.00	1.00 ^a^ ± 1.00	1.00 ^a^ ± 0.00	−0.0361
Cd, mg 100 g^−1^	ND	ND	ND	ND	-
Pb, mg 100 g^−1^	ND	ND	ND	ND	-

Mean ± standard deviation. ^a–c^ Means with different letters in rows indicate statistically significant differences at *p* < 0.05. *r*—correlation coefficient. *—statistically significant at *p* < 0.05. CC—control cheese made from CM milk (*n* = 12). C30—cheese from M30 milk (*n* = 12). C35—cheese from M35 milk (*n* = 12). C40—cheese from M40 milk (*n* = 12). ND—not detected.

**Table 4:** Physicochemical and texture properties of acid-curd cheese.

Properties	Cheese Type				*r*
	CC	C30	C35	C40	
pH	4.63 ^a^ ± 0.02	4.81 ^b^ ± 0.01	4.83 ^b^ ± 0.02	4.83 ^b^ ± 0.02	0.9861 *
Titratable acidity, °SH	58.23 ^c^ ± 1.07	52.41 ^b^ ± 0.62	48.82 ^a^ ± 0.56	48.38 ^a^ ± 0.10	−0.9668 *
Protein retention, %	73.86 ^a^ ± 0.08	75.44 ^b^ ± 0.12	75.85 ^c^ ± 0.07	76.51 ^d^ ± 0.26	0.9759 *
Fat, g 100 g^−1^	20.00 ^c^ ± 0.87	19.50 ^b^ ± 0.50	15.67 ^a^ ± 0.29	15.07 ^a^ ± 0.31	−0.7674 *
Moisture, g 100 g^−1^	57.39 ^a^ ± 0.11	61.48 ^b^ ± 0.85	66.81 ^c^ ± 1.60	70.67 ^d^ ± 0.57	0.8801 *
Hardness, N	3.61 ^c^ ± 0.18	3.24 ^c^ ± 0.20	2.45 ^b^ ± 0.05	1.95 ^a^ ± 0.21	−0.8285 *
Cohesiveness	0.23 ^c^ ± 0.01	0.20 ^c^ ± 0.02	0.10 ^b^ ± 0.02	0.05 ^a^ ± 0.03	−0.8174 *
Springiness, mm	3.40 ^c^ ± 0.06	2.13 ^b^ ± 0.20	1.97 ^b^ ± 0.17	1.06 ^a^ ± 0.06	−0.9441 *
Adhesiveness, mJ	4.30 ^a^ ± 0.35	5.60 ^b^ ± 0.26	5.70 ^b^ ± 0.10	5.70 ^b^ ± 0.10	−0.9395 *

Mean ± standard deviation. ^a–d^ Means with different letters in rows indicate statistically significant differences at *p* < 0.05. *r*—correlation coefficient. *—statistically significant at *p* < 0.05. CC—control cheese made from CM milk (*n* = 12). C30—cheese from M30 milk (*n* = 12). C35—cheese from M35 milk (*n* = 12). C40—cheese from M40 milk (*n* = 12).

**Table 5:** Organoleptic evaluation of acid-curd cheese.

Properties	Cheese Type				*r*
	CC	C30	C35	C40	
Overall acceptability	4.90 ^b^ ± 0.10	4.86 ^a, b^ ± 0.17	4.86 ^a, b^ ± 0.17	4.51 ^a^ ± 0.15	−0.8430 *
Apperance	4.80 ^b^ ± 0.20	4.60 ^a, b^ ± 0.41	4.60 ^a, b^ ± 0.29	4.25 ^a^ ± 0.48	−0.6214 *
Consistency	4.90 ^b^ ± 0.10	4.80 ^b^ ± 0.25	4.80 ^b^ ± 0.41	4.40 ^a^ ± 0.41	−0.6429 *
Taste	4.50 ^a^ ± 0.20	4.80 ^b^ ± 0.15	4.80 ^b^ ± 0.15	4.75 ^a^ ± 0.29	0.6956 *
Odour	5.00 ^a^ ± 0.00	4.88 ^a^ ± 0.25	4.90 ^a^ ± 0.25	4.88 ^a^ ± 0.25	−0.2515

Mean ± standard deviation. ^a–b^ Means with different letters in rows indicate statistically significant differences at *p* < 0.05. *r*—correlation coefficient. *—statistically significant at *p* < 0.05. CC—control cheese made from CM milk (*n* = 40). C30—cheese from M30 milk (*n* = 40). C35—cheese from M35 milk (*n* = 40). C40—cheese from M40 milk (*n* = 40).

**Table 6:** Physicochemical properties of goat acid whey.

Properties	Whey Type				*r*
	CW	W30	W35	W40	
pH	4.72 ^a^ ± 0.01	4.87 ^b^ ± 0.01	4.88 ^b, c^ ± 0.01	4.89 ^c^ ± 0.01	0.8939 *
Titratable acidity, °SH	37.40 ^b^ ± 0.20	35.00 ^a^ ± 0.20	35.07 ^a^ ± 0.83	34.27 ^a^ ± 0.76	0.9897 *
Protein, g 100 g^−1^	1.05 ^c^ ± 0.01	0.99 ^b^ ± 0.01	0.97 ^b^ ± 0.01	0.94 ^a^ ± 0.01	−0.9173 *
Fat, g 100 g^−1^	0.25 ^a^ ± 0.01	0.39 ^b^ ± 0.01	0.43 ^c^ ± 0.01	0.60 ^d^ ± 0.01	−0.9751 *
Lactose, g 100 g^−1^	4.07 ^b^ ± 0.01	4.06 ^b^ ± 0.01	4.06 ^b^ ± 0.01	3.85 ^a^ ± 0.01	0.8876 *

Mean ± standard deviation. ^a–d^ Means with different letters in rows indicate statistically significant differences at *p* < 0.05. *r*—correlation coefficient. *—statistically significant at *p* < 0.05. CW—control from CM milk (*n* = 12). W30—whey from M30 (*n* = 12). W35—whey from M35 (*n* = 12). W40—whey from M40 (*n* = 12).

**Table 7:** Mineral composition of goat whey.

Mineral Compound	Whey Type				*r*
	CW	W30	W35	W40	
Ca, mg 100 g^−1^	156.15 ^a^ ± 0.11	188.08 ^b^ ± 1.62	206.54 ^c^ ± 2.95	212.06 ^d^ ± 2.09	0.8939 *
K, mg 100 g^−1^	197.23 ^a^ ± 2.26	198.54 ^a^ ± 1.06	199.30 ^a^ ± 2.08	200.06 ^a^ ± 2.89	0.4822
Mg, mg 100 g^−1^	24.23 ^a^ ± 0.41	24.57 ^a^ ± 0.18	25.59 ^a^ ± 0.03	25.60 ^a^ ± 0.33	0.5911
Mn, µg 100 g^−1^	5.00 ^a^ ± 1.01	5.00 ^a^ ± 1.02	4.00 ^a^ ± 1.03	4.00 ^a^ ± 1.01	−0.4153
P, mg 100 g^−1^	96.59 ^a^ ± 1.24	96.52 ^a^ ± 1.66	95.39 ^a^ ± 3.77	95.24 ^a^ ± 1.74	0.3318
Se, µg 100 g^−1^	ND	ND	1.00 ^a^ ± 0.00	1.00 ^a^ ± 0.00	0.2679
Cd, mg 100 g^−1^	ND	ND	ND	ND	-
Pb, mg 100 g^−1^	ND	ND	ND	ND	-

Mean ± standard deviation. ^a–d^ Means with different letters in rows indicate statistically significant differences at *p* < 0.05. *r*—correlation coefficient. *—statistically significant at *p* < 0.05. CW—control from CM milk (*n* = 12). W30—whey from M30 (*n* = 12). W35—whey from M35 (*n* = 12). W40—whey from M40 (*n* = 12). ND—not detected.
